# Nerve injury induces robust allodynia and ectopic discharges in Na_v_1.3 null mutant mice

**DOI:** 10.1186/1744-8069-2-33

**Published:** 2006-10-19

**Authors:** Mohammed A Nassar, Mark D Baker, Alessandra Levato, Rachel Ingram, Giovanna Mallucci, Stephen B McMahon, John N Wood

**Affiliations:** 1 Molecular Nociception Group, Department of Biology, University College London WC1E 6BT, UK; 2 Centre for Neuroscience Research, Kings College London, London SE1 7EH, UK; 3 MRC Prion Unit and Department of Neurodegeneration. Institute of Neurology, Queen Square, London WC1N 3BG, UK

## Abstract

Changes in sodium channel activity and neuronal hyperexcitability contribute to neuropathic pain, a major clinical problem. There is strong evidence that the re-expression of the embryonic voltage-gated sodium channel subunit Na_v_1.3 underlies neuronal hyperexcitability and neuropathic pain.

Here we show that acute and inflammatory pain behaviour is unchanged in global Na_v_1.3 mutant mice. Surprisingly, neuropathic pain also developed normally in the Na_v_1.3 mutant mouse. To rule out any genetic compensation mechanisms that may have masked the phenotype, we investigated neuropathic pain in two conditional Na_v_1.3 mutant mouse lines. We used Na_v_1.8-Cre mice to delete Nav1.3 in nociceptors at E14 and NFH-Cre mice to delete Na_v_1.3 throughout the nervous system postnatally. Again normal levels of neuropathic pain developed after nerve injury in both lines. Furthermore, ectopic discharges from damaged nerves were unaffected by the absence of Na_v_1.3 in global knock-out mice. Our data demonstrate that Na_v_1.3 is neither necessary nor sufficient for the development of nerve-injury related pain.

## Background

Neuropathic pain is an important and unmet clinical problem. There is a lack of effective drugs for its treatment and in some cases the pain is resistant to the strongest analgesics [1]. Hyperexcitability of damaged sensory neurons, as a result of injury to and/or demyelination of their peripheral axons, is thought to initiate the processes that lead to most neuropathic pain [2,3]. Hyper-excitable neurons can fire spontaneously, causing spontaneous pain, or become hypersensitive to otherwise innocuous mechanical and thermal stimuli giving rise to allodynia (noxious responses to innocuous stimuli) and hyperalgesia (increased perception of noxious stimuli) [2,3].

Injured sensory neurons undergo major changes in gene expression that have been catalogued in microarray studies [4-6]. Altered expression of voltage-gated sodium channels (VGSC), which underlie the electrical excitability of nerve and muscle, has been extensively studied [7]. Ectopic activity in damaged neurons [8] and neuropathic pain behavior [9] have been shown to be sensitive to the VGSC blocker, Tetrodotoxin (TTX). Sensory neurons express multiple subtypes of TTX-sensitive and TTX-resistant VGSCs [10]. The expression of Na_v_1.1, Na_v_1.2, Na_v_1.6, Na_v_1.7, Na_v_1.8 and Na_v_1.9 subunits is down-regulated, whereas only Na_v_1.3, a TTX-sensitive channel, is up-regulated following peripheral nerve injury [10-16]. Na_v_1.3 is expressed throughout the embryonic nervous system but is down-regulated in adults [11,17]. Coinciding with the re-expression of the Na_v_1.3 channel in injured neurons, the voltage-gated sodium currents recover from inactivation fourfold faster than that in uninjured neurons [13]. This is thought to be a direct result of expression of Nav1.3 as it possesses similar inactivation kinetics when expressed in cell lines [18]. Rapid recovery from inactivation could allow damaged nerves to fire at higher frequencies than otherwise [13]. Therefore, it is hypothesized that mis-expression of VGSCs and in particular the re-expression of Nav1.3 is an important factor contributing to the hyperexcitability of injured neurons. Several pieces of evidence support this hypothesis. Firstly, the ectopic activity [8] and mechanical allodynia [9] associated with nerve injury has been shown to be sensitive to TTX. Secondly, Glial-derived neurotrophic factor (GDNF), which reverses neuropathic pain behaviour, reverses Na_v_1.3 up-regulation [19]. Finally, intrathecal administration of antisense oligonucleotides directed against Na_v_1.3 mRNA reverses neuropathic pain behaviour and restores the inactivation kinetics of VGSC to that of uninjured neurons [20,21]. Taken together, these data suggest that Na_v_1.3 is a good target for analgesic drug development [22]. However, a recent study using a different antisense oligonucleotide sequence directed against Na_v_1.3 failed to reverse neuropathic pain behaviour [23].

Here we describe the generation of 3 Na_v_1.3 mutant mouse lines to test the hypothesis that Na_v_1.3 has a causative role in nerve-injury-induced chronic pain [22]. Our data shows, contrary to expectation, that Na_v_1.3 is neither responsible nor necessary for ectopic activity and neuropathic pain behaviour.

## Results and Discussion

**Figure 1:**
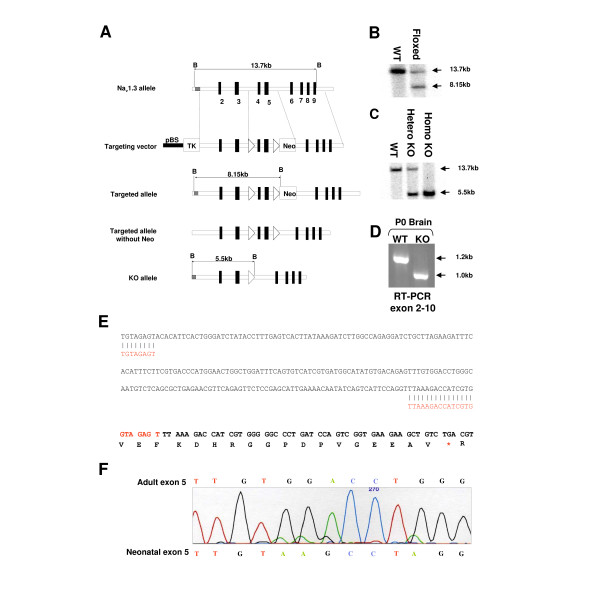
**Generation of floxed and global Na_v_1.3 KO mice**. (**A**) Schematic representation of Na_v_1.3 WT locus, targeting construct, floxed allele and global KO allele. (**B**) Southern blotting of tail DNA with BamHI and 5' probe confirms correct insertion of the targeting construct and (**C**) deletion of floxed exons in global KO mice. (**D**) RT-PCR on 1 μg of total RNA from P0 Brain confirms complete deletion of exon 4&5 in global KO mice. (**E**) Alignment of sequence of KO (red) and WT (black) RT-PCR bands shows the deleted 221 bp. Splicing of exon 3 (red) and 6 (black) causes frame shift and a truncated protein (asterisk = stop codon). (**F**) Sequencing of the WT RT-PCR shows that the P0 brain contains the two forms of exon five. The adult form is about 4 times more than the neonatal form.

We used a genetic approach to investigate the contribution of Na_v_1.3 to setting pain thresholds, to the hyperexcitability of injured neurons and neuropathic pain behaviour in mice. We generated a floxed Na_v_1.3 mouse, (Figure 1a–b) and used a deletor mouse strain expressing Cre recombinase before E4 [24] to generate a conventional global null mutant (Na_v_1.3 KO), (Figure 1c). To confirm deletion of Na_v_1.3 we used RT-PCR to amplify sequences between exons 2 and 10, figure 1d. Sequencing of the PCR product from the Na_v_1.3 KO brain confirmed the deletion of 221 bp representing the floxed exons 4 and 5, (Figure 1e). Sequencing of the PCR product from WT brain revealed that most of the Na_v_1.3 mRNA carries the adult exon five (about 80%), (Figure 1f).

**Figure 2:**
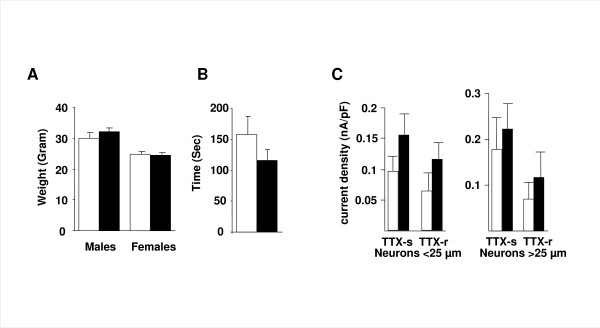
**Global Na_v_1.3 KO mice develop normally**. (**A**) Weight (males *P *= 0.28 and females *P *= 0.80) and (**B**) performance on the rotarod (*P *= 0.21) were not different between the Na_v_1.3 KO (black) and WT (white). (**C**) Peak current density (nA/pF) of TTX-s current for small neurons (<25 μm) in WT = 0.097 ± 0.023 *vs*. KO = 0.156 ± 0.033 (*n *= 9 and 11, respectively; *P *= 0.16). TTX-r current (corresponding to Na_v_1.8) in WT = 0.065 ± 0.028 *vs*. KO = 0.115 ± 0.029 (*P *= 0.24). Peak current density of TTX-s current in medium neurons (>27 μm) in WT = 0.179 ± 0.69 *vs*. KO = 0.223 ± 0.055 (*n *= 5 and 6, respectively; *P *= 0.63). TTX-r current in WT = 0.071 ± 0.034 *vs*. KO = 0.118 ± 0.054 (*P *= 0.49).

Na_v_1.3 KO mice were healthy, fertile, grew as well as their littermate controls (WT), (figure 2a), and performed equally well on the rotarod, (figure 2b). Peak sodium currents in cultured sensory neurons were unaffected by Na_v_1.3 gene deletion, (figure 2c).

**Figure 3:**
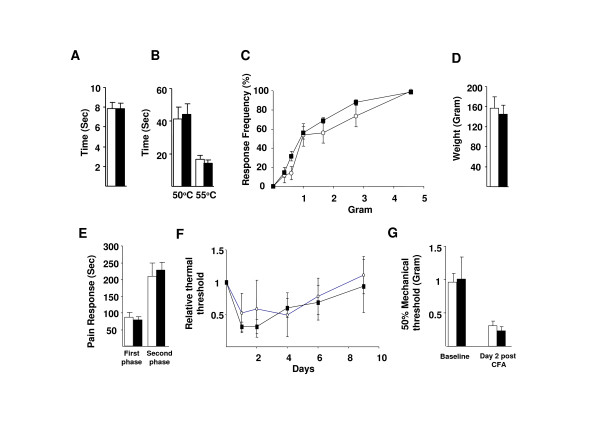
**Acute and inflammatory pain behaviour in Na_v_1.3 KO is normal**. Thermal threshold in the Hargreave's (**A**, WT 7.83 ± 0.7 sec, n = 8; KO 7.82 ± 0.6 sec, n = 11; *P *= 0.9) and hotplate apparatus (**B**, at 50°C WT 41.3 ± 7.3, n = 13; KO 44.2 ± 6.5, n = 13; *P *= 0.7. at 55°C WT 16.6 ± 2.4; KO 14.3 ± 1.8; *P *= 0.1) were similar. Mechanical threshold to range of von Frey hairs applied to hindpaw (**C**) and plunt force applied to tail (**D**, WT 157 ± 22.7 g, n = 13; KO 145 ± 18.2, n = 13; *P *= 0.4) were not different. Both phase of the formalin pain response were not different (**E**, phase 1 WT 86.1 ± 12.9 sec, n = 8; KO 78.4 ± 9.2, n = 8; *P *= 0.6. Phase 2 WT 210 ± 38.3 sec; KO 227.4 ± 24.2; *P *= 0.7). Injection of CFA in hindpaw elicited thermal (**F**) and mechanical hyperalgesia 2 days post injection (**G**, WT 0.3 ± 0.08 gram, n = 6; KO 0.23 ± 0.05, n = 6; *P *= 0.5) to the same level in both mice groups.

We next examined acute pain thresholds in adult Na_v_1.3 KO animals. Thermal and mechanical thresholds are normal, (Figure 3a–d), indicating that Na_v_1.3 does not play a role in setting pain thresholds in the undamaged nervous system. Although it has been reported that Na_v_1.3 is up-regulated in an inflammatory pain model [25], we found that pain behaviour evoked by injection of formalin and CFA into the hindpaw was the same in both Na_v_1.3 KO and WT mice, (Figure 3e–g). This indicates that Na_v_1.3 does not play a role in inflammatory pain, in marked contrast to Na_v_1.7 KO mouse, which shows an almost complete loss of inflammatory pain [26].

### Nav1.3 and neuronal excitability

**Figure 4:**
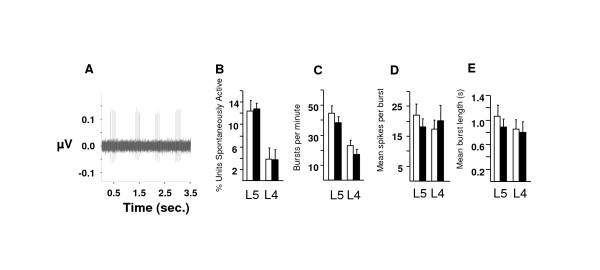
**Ectopic discharges is unchanged in Na_v_1.3 KO mouse**. Ectopic discharges in L5 (cut) and L4 (spared) sensory neurons following nerve injury are not different in the Na_v_1.3 KO global mouse (black). (**A**) An example trace from spontaneously active L5 fibres from Na_v_1.3 KO mouse. (**B**) Number of spontaneously active fibres in L5 is 3 times greater than that in L4 (L5 *vs*. L4 in WT *P *= 0.016 and in KO *P *= 0.016). Spontaneous activity is not different between KO and WT (*P *= 0.89 in L5 and *P *= 0.98 in L4, n = 6 mice in all groups). (**C**) Mean number of burst per minute in L5 is double that in L4 (L5 *vs*. L4 in WT *P *= 0.025 and in KO *P *= 0.022) but not different between KO and WT (*P *= 0.37 in L5 and *P *= 0.33 in L4). (**D**) Mean number of spikes per burst is not different between KO and WT (*P *= 0.58 in L5 and *P *= 0.61 in L4) and between L5 and L4 (WT *P *= 0.95 and in KO *P *= 0.58). (**E**) Mean burst length is not different between KO and WT (*P *= 0.63 in L5 and *P *= 0.96 in L4) and between L5 and L4 (WT *P *= 0.73 and in KO *P *= 0.56).

Does Na_v_1.3 play a role in neuronal hyperexcitabtility following nerve injury? Voltage-gated sodium currents in injured neurons recover from inactivation fourfold faster than that in naive neurons possibly as a result of up-regulation of Na_v_1.3 [13,18]. Rapid recovery from inactivation could allow damaged nerves to fire at higher frequencies [11]. The ectopic activity associated with nerve injury, which has been shown to be sensitive to TTX, is an important trigger for neuropathic pain behaviour [8,27]. We therefore measured ectopic discharges from teased fibres of the damaged (L5) and spared (L4) 24 hours after surgery in Na_v_1.3 KO and WT mice. L5 spinal nerve ligation induces spontaneous activity in L4 as well as L5 myelinated fibres, though the percentage of active neurons in mouse appears to be less than that in rat [19]. The percentage of spontaneously active fibres from L5 DRG was three fold higher than that from L4 DRG, (Figure 4b), which is similar to earlier results in rats [19]. Importantly, the percentage of spontaneously active fibres was not different between the Na_v_1.3 KO and WT, (Figure 4a). In addition, other characteristics of the spontaneous activity such as the number of bursts per minute, (Figure 4b), mean number of spikes per burst, (Figure 4c), and the mean burst length, (Figure 4c), were also not different. These findings suggest that Na_v_1.3 does not play an essential role in the ectopic hyperexcitability of damaged nerves.

### Nav1.3 and neuropathic pain

**Figure 5:**
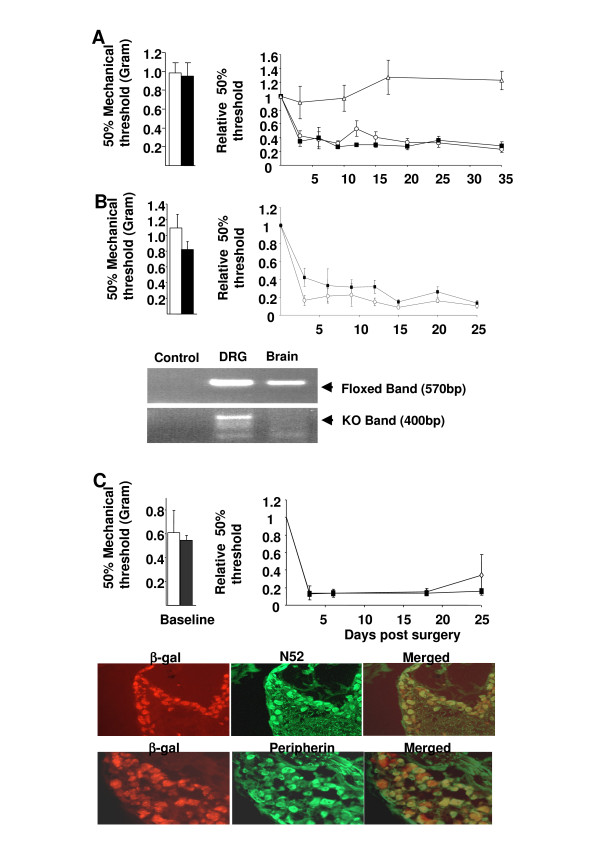
**Neuropathic pain behaviour is unaffected in global and conditional Na_v_1.3 KO mice**. Robust mechanical allodynia developed in both global (**A**), nociceptor-specific (**B**) and postnatal KO (**C**) of Na_v_1.3 but not in sham operated WT (white triangle in **A**). The histogram to the left shows the baseline before injury. Nociceptor-specific deletion of Na_v_1.3 was confirmed by PCR on genomic DNA (in **B**) which shows a product for the floxed Nav1.3 allele in both DRGs and brain. However, only DRGs produce the KO allele. Note that non-neuronal cells in DRG will contribute to the floxed Nav1.3 band. In **C **Co-staining in sections from adult DRG from NFH-Cre/Rosa26 mouse shows that most sensory neurons express Cre in NFH-Cre mouse.

GDNF reverses the up-regulation of Na_v_1.3 and neuropathic pain behaviour [19], and antisense oligonucleotides against Na_v_1.3 sequence reverse neuropathic pain behaviour [20,21]. Therefore, we measured mechanical pain thresholds in Na_v_1.3 KO mice following tight ligation of spinal nerve L5 (Chung model). Surprisingly, a robust mechanical allodynia developed in both Na_v_1.3 KO and WT mice from day 3 post-surgery and persisted throughout the experiment, (Figure 5a). Sodium currents in cultured sensory neurons were unaffected by Na_v_1.3 gene deletion, (Figure 2c), suggesting a lack of compensatory changes.

In order to rule out the possibility that genetic compensation had confounded the behavioural phenotype in the Na_v_1.3 KO, we generated two conditional knockout strains; we mated the floxed Na_v_1.3 mouse to the Na_v_1.8 Cre line [28] to delete Na_v_1.3 in nociceptors from E14, and with the NFH-Cre mouse line [29] to delete Na_v_1.3 in CNS and PNS from the 10^th ^postnatal week. Mice in both conditional knockout strains developed a profound mechanical allodynia soon after surgery that was not different from that of littermate controls (floxed Na_v_1.3), (Figure 5b and 5c), confirming our results using the conventional global KO. We conclude that Na_v_1.3 does not play a critical role in either the induction or maintenance of neuropathic pain behaviour.

Our results can be explained as follows; the level of Na_v_1.3 expression in sensory neurons following injury is estimated to increase by about 1.4–2 fold [5,15] which is still very small considering the almost undetectable level of Na_v_1.3 in adult sensory neurons. Thus the increase in sodium currents due to the up-regulated Na_v_1.3 is unlikely to be significant in comparison to the total sodium current before injury. The reported fast kinetics of the Na_v_1.3 current [18] suggests that the qualitative aspect of the Na_v_1.3 up-regulation may be more important than the quantitative one. Our results using the global Na_v_1.3 KO rule out this in stimulus-evoked neuropathic pain. This is because Na_v_1.3 is deleted in all cell types regardless of the extent to which they up-regulate Na_v_1.3, the type of cells involved (small vs large sensory neurons and DRG vs central neurons) and the sub-cellular localization of Na_v_1.3 (somata vs terminals). Furthermore, although some antisense studies suggest a causal role for Na_v_1.3 in neuropathic pain [20,21], the specificity of the oligonucleotides used is not absolute (of 21 bases 15 are identical to Na_v_1.1, 17 to Na_v_1.2 and 14 to Na_v_1.6), and another antisense study using different specific oligonucleotides has failed to confirm these findings [23].

### Sodium channels and neuropathic pain

The role of VGSCs in the pathogenesis of neuropathic pain has recently come under increasing scrutiny. Changes in neuronal hyperexcitability have not been found to correlate with changes in sodium currents [30]. Nevertheless, the effectiveness of non-subtype selective sodium channel blockers in treating neuropathic pain [1] has stimulated the search for a specific underlying sodium channel subunit against which, more potent and selective analgesics can be developed. It seems likely, however, that no single sodium channel is specifically associated with neuronal hyperexcitability in damaged nerves, but that aberrant expression of high densities of sodium channels at the damaged terminals of sensory neurons may result in hyperexcitability [31]. In support of this we have recently shown that neither Na_v_1.7 nor 1.8 is necessary for the development of neuropathic pain [32]. Na_v_1.9 null mutants have also been shown to develop neuropathic pain normally [33]. By contrast, it is clear that Na_v_1.7 plays a major role in the development of inflammatory pain and subtype specific channel blockers directed against this channel may be desirable for the treatment of inflammatory pain.

The role in neuropathic pain pathogenesis of the other major VGSC subunits in DRG, Na_v_1.1, Na_v_1.2 and Na_v_1.6, remains to be investigated. Because of their relative abundance it is expected that deletion of these subunits may not have a specific effect on neuropathic pain. Almost certainly the usefulness of Na_v_1.1, Na_v_1.2 or Na_v_1.6 blockers in treating neuropathic pain will be greatly undermined by their broad expression in the CNS [10].

## Conclusion

Neuropathic pain may be more amenable to local treatment with broad-spectrum sodium channel blockers, rather than those targeted at a specific sodium channel subunit such as Na_v_1.3.

## Methods

### Generation of floxed Na_v_1.3 mouse

An RCPI-22 129S6/SvEvTac mouse BAC library was screened using a probe representing exons 4–12. DNA from positive BAC clones was prepared and subcloned into pBluescript (BS-SKII-). Three subclones were obtained that covered Na_v_1.3 sequences from exon 2–8. The construct was prepared in three steps. Firstly, the 3' arm, Neomycin cassette and one LoxP site were cloned together as follows; two oligonucleotides were cloned in BS-SKII- to form new cloning sites. One LoxP site was cloned as an EcoRI-PstI fragment from PLneo vector [26]. The 3' arm was cloned as ClaI-BamHI 7 Kb fragment. Neomycin cassette was cloned as BamHI-SalL fragment from Py6.0 plasmid [26]. Secondly, the 5' arm, one LoxP and the floxed exons were cloned together as follows; 7 kb fragment ClaI-SacI was cloned in BS-SKII-. A LoxP site was inserted into the BsmBI site of the genomic fragment through blunt ligation. Finally, the two halves above were cloned into a vector containing the Thymidine-Kinase (TK) cassette as follows; the 3' side was prepared as Not-ClaI fragment, the 5' side was prepared as ClaI-SacII fragment and the TK containing vector was prepared as Not-SacII fragment. All were ligated together in the same tube. Positive clones were confirmed by restriction digests and sequencing. Blastocyst injection was performed by Monica Mendelson (Colombia University). Chimeras were crossed to C57BL/6 and germ line transmission tested using Southern blotting of tail DNA derived from F1 offspring. F1 heterozygotes were crossed to FLPe deletor animals [34] to excise the positive selection marker.

### Behavioural analysis

All tests were approved by the United Kingdom Home Office Animals (Scientific Procedures) Act 1986. They were performed in a Home Office designated room at 22 ± 2°C. Tests were carried out as previously descried [28]. Formalin test: 20 μl of 5% formalin (37% formaldehyde (BDH) diluted with 0.9% saline (Sigma)) was injected subcutaneously into the plantar aspect of a hind-paw using a Hamilton syringe and 27 g needle and animals were placed in a 20 × 20 × 20 cm Plexiform chamber. The amount of time spent licking or biting the injected paw was then recorded in 5 minute periods over the next hour. Animals were habituated to the test environment for at least 1 hr before experiments were started. Activity during the first phase, from 0–10 mins, and the second phase, from 10–60 mins, was then calculated for each animal.

### Induction of Neuropathic pain

Neuropathic pain was induced according to the Chung Model (ligation of spinal nerve L5). Baseline mechanical thresholds for one paw were measured from mice using von Frey hairs (using the up-down method) respectively. Animals were anaesthetised using Halothane. A midline incision was made in the skin of the back at the L_2_-S_2 _levels and the left paraspinal muscles separated from the spinal processes, facet joints and transverse processes at the L_4_-S_1 _levels. The L_5 _transverse process was removed and the L_5 _spinal nerve tightly ligated using 8-0 silk thread.

### PCR on Genomic DNA

DNA was precipitated from DRG and brain after overnight incubation in lysis-buffer (100 mM Tric-Cl, 5 mM EDTA, 0.2% SDS, 200 mM NaCl and 0.1 mg/ml Proteinase K). DNA pellet was resuspended in 200 μl TE and 1 μl was used for each 25 μl PCR reaction. Nav1.3 Primers used are (GAGAGAAAGACACTTAAATGCAGACATC), (GCTTTTTGTTCAAGTCTATCATATTCAAAG) and (AAG GAT GGC ATC ACC CAC AAG).

### RT-PCR

Deletion of exons 4 and 5 was confirmed by reverse transcription PCR (RT-PCR). RNA isolation was performed using TRIzol Reagent (Invitrogen, Paisley, UK), according to manufacturer's protocol. The reverse transcription reaction was also performed according to an Invitrogen protocol, using random primers. Primers used were (CACTAAACCCCGTTAGGAAAATTGCT) and (TCCAAGTGCTCCCTCTGTCTCCTC). The same primers were used for sequencing the RT-PCR bands.

### Immunohistochemistry

Animals were terminally anaesthetised with 150 mg/kg sodium pentobarbitone (Rhône Mérieux), and then intracardially perfused with ice cold PBS (0.14 M NaCl, 2.7 mM KCl, 10 mM Na_2_HPO_4_, 1.7 mMKH_2_PO_4_) followed by ice cold 4% paraformaldehyde/PBS. The tissues were removed and placed in O.C.T compound. 10 μm sections were prepared and mounted on Superfrost Plus slides (BDH). Sections were fixed in 4% paraformaldehyde for 5 mins and washed 3 × 10 mins in PBS + 0.1% Triton-X. They were blocked and permeabilised in 10% goat serum (GibcoBRL)/PBS + 0.1% Triton-X for at least 3 hrs at room temperature. Sections were then incubated in the following antisera in 10% goat serum/PBS + 0.3% Triton-X overnight at 4°C: rabbit anti-beta-galactosidase (5'-3') (1:500), mouse anti-peripherin (Chemicon) (1:1000) or mouse anti-neurofilament (Sigma N-52) (1:1000). They were then washed 3 × 10 mins in PBS + 0.1% Triton-X. The primary antisera were localised by immunofluorescence with Alexa Fluor 594 (Molecular Probes) (1:1000) and Alexa Fluor 488 (Molecular Probes) (1:1000). The slides were finally washed 3 × 30 mins at 4°C and mounted using Aqueous Mounting Solution (Sigma).

### Measuring Peak sodium current

Cultures of dorsal root ganglion (DRG) neurons were prepared from adult Na_v_1.3 null and WT littermate mice. The animals were killed by cervical dislocation, and dorsal root ganglia (DRG) were removed and enzymatically dissociated (Dispase/collagenase; Sigma, Poole, Dorset, UK). The isolated and pooled DRG neurons were plated onto poly-L-lysine coated glass coverslips and maintained in culture for 1–2 days.

### Electrophysiology and solutions

Conventional voltage-clamp recordings in the whole-cell patch-clamp configuration were made from neurones of varying sizes (corresponding to small and medium sized neurons with a maximum apparent diameter of 43 μm) using an Axopatch 200B amplifier (Axon Instruments, Union City, California, USA) driven from a PC generating pulse protocols (Pclamp 9, Axon Instruments). The neuronal capacitance was estimated through the procedure of capacity transient cancellation, soon after attaining the whole-cell configuration. Electrodes were made from thin-walled glass (Harvard Apparatus, Edenbridge, Kent, UK) and had an initial resistance of between 1.5 and 2 MΩ when filled with intracellular solution. Series-resistance compensation was set near 70% with a nominal feed-back lag of 12 ms. Recordings were filtered at 5 KHz (4-pole Bessel filter) and sampled at 10 KHz. Recordings were made at room temperature.

TTX-s and TTX-r Na^+ ^currents were recorded in relative isolation by including pharmacological blockers of both K^+ ^and Ca^2+ ^currents in both extracellular and intracellular media. In order to distinguish TTX-s and TTX-r currents, recordings were made before and after the addition of 250 nM TTX to the extracellular solution, where the TTX-s component was subsequently derived by off-line analysis using digital subtraction. The extracellular solution contained (in mM): NaCl 43.3, Tetraethylammonium Chloride 96.7, HEPES 10, CaCl_2 _2.1, MgCl_2 _2.12, 4-Aminopyridine 0.5, CsCl 10, KCl 7.5, CdCl_2 _0.1. The intracellular solution contained (in mM): CsCl 130, CsF 13, EGTA (Na) 3, Tetraethylammonium Chloride 10, HEPES 10, CaCl_2 _1.21, ATP(Mg) 3 mM, GTP (Li) 500 μM. External and internal solutions were buffered to pH 7.2–7.3 with the addition of CsOH. Tetrodotoxin (TTX, 250 nM) was locally applied and removed by gravity fed superfusion. The reagents, with the exception of TTX, were obtained from Sigma-Aldrich (Poole, Dorset, UK). TTX was purchased from Alomone Labs (TCS Biologicals, Botolph Claydon, Bucks, UK).

In order to derive current density measurements, peak Na^+ ^currents were recorded in response to a family of incrementing, depolarizing clamp-steps, filtered at 5 KHz (4-pole Bessel filter) and normally sampled at 20 KHz. The holding potential was -80 mV, and the clamp-steps were preceded by a 20 ms pre-pulse. These values of membrane potential, coupled with the presence of internal F^-^, were not optimal for recording TTX-r persistent Na^+ ^current, and the TTX-r currents analysed always exhibited the voltage-dependent and kinetic characteristics of Na_v_1.8. Current density estimates were found by dividing the peak-current values by the value estimated for membrane capacitance. TTX-s current amplitudes were found by off-line digital subtraction.

### Recording of spontaneously active fibres

At defined time points after L5 spinal nerve ligation mice were anaesthetised via a brief induction with isofluorane gas followed by an i.p. injection of pentobarbitone (100 mg/kg). A lumbar laminectomy was performed and the L4 and L5 dorsal roots were identified and cut close to the spinal cord. Both the ventral roots and the L4 spinal nerve were cut as far distally as possible and then the dorsal roots, ventral roots, DRGs and spinal nerves (in continuity) from L4 and L5 were carefully and quickly removed. Throughout the process of dissection the tissue was kept hydrated by regularly applying oxygenated (95% O_2_, 5% CO_2_) aCSF solution (in millimolar: NaCl 118; KCl 4.7; MgSO_4 _1.2; KH_2_PO_4 _1.2; NaHCO_3 _25; CaCl 2.5 and glucose 11) to the preparation. The tissue was then kept in oxygenated aCSF at room temperature for 30 minutes before the start of electrophysiological recording. The peripheral nerve preparations were mounted in a two chamber recording bath with the spinal nerve and DRG on one side continuously perfused with oxygenated aCSF at a rate of 10 ml/min. This solution was kept at 35 ± 1°C by running the perfusion tube through a jacket containing water heated and circulated by a thermostatically controlled water bath. The dorsal and ventral roots were sealed in the other chamber and covered in mineral oil which had been previously oxygenated with an air tube. Oil in this chamber was periodically replaced to maintain hydration and oxygenation of the dorsal root. A leak-proof partition barrier of perspex and high-vacuum grease ensured electrical isolation from the other compartment. For the purposes of electrical stimulation the end of the spinal nerve was taken up into a suction electrode with bipolar silver/silver chloride wires. Fine filaments were teased from the dorsal root using sharpened watchmakers forceps. These strands were placed over electrodes made of sliver wire coated with teflon that had been stripped away from the tip. A crushed strand from the ventral root was placed across another silver wire electrode to act as an indifferent recording. Signals from the electrodes were passed through a head-stage which was also connected to earth. From here the signal was amplified and filtered (low pass 500 Hz, high pass 5 KHz) using Neurolog equipment (Digitimer, UK). Residual 50 Hz noise was removed with a Humbug noise eliminator (Quest Scientific, Canada). The signal was viewed on a digital oscilloscope (Tektronix, USA) and also recorded for off-line analysis (Powerlab and Chart 5 software, ADI instruments, UK).

Spontaneously active units were recorded in both the L5 and L4 dorsal roots. In strands with multiple ectopically firing units separation was achieved on the basis of differing amplitude, action potential shape and interspike interval. The number of conducting fibres in each strand was determined by incremental electrical stimulation of the spinal nerve whilst observing the recruitment of units on a storage oscilloscope (Gould, UK). Strands that were studied typically contained a total of 8 to 12 conducting units and around 50 to 60 units were sampled from each dorsal root. The proportion of spontaneously active units in each preparation was calculated as a percentage and then a mean obtained for all data in a group with n representing a single L4 or L5 root taken from one animal. A large proportion of units observed firing spontaneously did so in a bursting manner and so further analysis of these patterns was undertaken using specialised software (Spike 2, Cambridge Electronic Design, UK).

### Statistics

Unpaired t-test was used to compare data unless otherwise indicated.

## Competing interests

The author(s) declare that they have no competing interests.
